# Polymorphisms in *IL-10* and *TGF-β* gene promoter are associated with lower risk to gastric cancer in a Mexican population

**DOI:** 10.1186/s12885-019-5627-z

**Published:** 2019-05-15

**Authors:** Cecilia Martínez-Campos, Kirvis Torres-Poveda, Margarita Camorlinga-Ponce, Lourdes Flores-Luna, Carmen Maldonado-Bernal, Vicente Madrid-Marina, Javier Torres

**Affiliations:** 1 Department of Molecular Genetics and Microbiology, Duke University Medical Center, Durham, NC USA; 2 Dirección de Infecciones Crónicas y Cáncer. Centro de Investigación sobre Enfermedades Infecciosas, Instituto Nacional de Salud Pública, Cuernavaca, Morelos Mexico; 3 CONACyT-Instituto Nacional de Salud Pública, Cuernavaca, Morelos Mexico; 4 Unidad de Investigación en Enfermedades Infecciosas, UMAE Pediatría, CMN S-XXI, IMSS, Mexico City, Mexico; 5 Centro de investigación en Salud Poblacional, Instituto Nacional de Salud Pública, Cuernavaca, Morelos Mexico; 6 Laboratorio de Investigación en Inmunología y Proteómica, Hospital Infantil de Mexico Federico Gómez, SS, Mexico City, Mexico

**Keywords:** Single nucleotide polymorphism, Cytokines, *H*, *Pylori*, Gastric cancer

## Abstract

**Background:**

*Helicobacter pylori* infection is recognized as the main risk factor for gastric cancer (GC), the fifth most common neoplasia worldwide. *H. pylori* interacts with the immune system, disrupting the cytokine network and inducing chronic inflammation. This work aimed to evaluate the association between single nucleotide polymorphisms (SNPs) in selected cytokine gene promoters and GC.

**Methods:**

The study included 359 subjects, 125 GC patients, 109 intestinal metaplasia (IM) patients and 125 asymptomatic controls. DNA was extracted from white blood cells and nine SNPs in cytokine gene promoters were genotyped using predesigned 5′-endonulease assays. The association of the SNPs with IM and GC was evaluated using multinomial regression models.

**Results:**

Both genotypes, TC (*OR* = 0.51, 95% CI = 0.27–0.98) and TT (*OR* = 0.42, 95% CI = 0.20–0.91) in the locus − 509 of the *TGF-β* promoter were significantly associated with GC. The TT genotype in the locus − 819 of the *IL-10* promoter was also significantly associated with GC (*OR* = 0.37, 95% CI = 0.17–0.81). No significant association was found with SNPs *IL-4* –590 T/C (rs1800629), *IL-6* –573G/C (rs1800796), *IL-10* –592C/A (rs1800872), *IL-10* –1082A/G (rs1800896), and, *IFN-γ* –1615C/T (rs2069705).

**Conclusions:**

SNPs in *TGFβ* (− 509 C/T, rs1800469) and *IL-10* (− 819 C/T, rs1800871) promoters were associated with a lower risk for GC in a Mexican population.

## Background

Gastric cancer (GC) is the fifth most common cancer type and the third leading cause of death by cancer worldwide [1]. The highest estimated mortality rates occur in Eastern Asia, Eastern Europe, and Central and South America [1], where cases usually seek medical attention at later stages when prognosis is very poor. In Mexico, GC is the sixth most common cancer after breast, prostate, cervix uteri, colorectal, and lung cancer, and represents the fifth cause of cancer deaths.

While several socio-demographic and environmental risk factors have been associated with GC, infection by *Helicobacter pylori* is recognized as the main risk factor [2], as it is classified as a group I carcinogen by the IARC (International Agency for Research on Cancer) [3]. Prevalence of *H. pylori* in countries with high GC incidence ranges from 31 to 73% in the general population, and from 11 to 66.2% in children [4].

The most widely accepted mechanism by which *H. pylori* contributes to carcinogenesis is the induction of a chronic and dysregulated inflammation; the immune response against this gram-negative bacterium may also contribute to its pathogenesis. The attachment of *H. pylori* to the gastric epithelial cells induces the release of inflammatory cytokines that recruit and activate T lymphocytes, macrophages, and plasma cells [5]. Cytokines have pleiotropic effects on immune and epithelial cells, regulating cell proliferation and differentiation and modulating the secretion of other cytokines and the type and degree of inflammation. A chronic long-lasting dysregulated inflammation in the gastric mucosa is recognized as the main driving mechanism to cause tissue and DNA damage that may lead to gastric cancer.

*H. pylori* infection is often acquired in childhood and colonization of the gastric epithelium promotes an upregulation of TLR-2, − 4, − 5, and − 9, and the expression of cytokines such as IL-1, IL-6, IL-8, TNF-α, IFN-γ, TGF-β, and IL-10 [6]. The type and amount of cytokines produced in response to *H. pylori* infection have a significant impact on the risk of developing GC. This may depend on the kind of cytokines released by different subsets of differentiated CD4^+^ helper T cells in response to *H. pylori*. The pattern of cytokines secreted by the T helper (Th) cells will vary depending on the subsets cells; Th1 cells secretes IFN-γ, whereas Th2, secretes IL4, IL-6, and IL-10. Th3 secretes TGF-β, Th17 secretes IL-17A and IL-22 and Th22 secretes IL-22 [7]. Accordingly, cytokines are obvious candidates to be studied as GC risk factors, and polymorphisms in several cytokine genes may influence the risk for GC.

Considering that cancer is a disease with a multifactorial origin and that GC is characterized by a disruption of the cytokine network induced by *H. pylori*, we hypothesized that some SNPs in cytokine gene promoters might be associated with the risk to intestinal metaplasia (IM) and GC.

## Methods

### Population under study

The population under study was selected as previously described [8], and similar procedures were followed for sample collection. Briefly, the population was selected from patients over 30 years of age seeking attention because of gastroduodenal symptoms at the Hospital de Oncología, Centro Médico Siglo XXI, Instituto Mexicano del Seguro Social (IMSS), and Instituto Nacional de Cancerología, Secretaría de Salud, Mexico City. Diagnosis was based on endoscopic examination and confirmed by histopathology studies, and patients with confirmed IM or GC diagnosis were invited to participate. Exclusion criteria were: patients under treatment for cancer or being administered with antibiotics, bismuth compounds, proton-pump inhibitors, or nonsteroidal anti-inflammatory drugs for at least 2 weeks prior to the study. The control group included healthy blood donors without any symptom or medication attending the blood bank of the Centro Médico Siglo XXI, IMSS, Mexico City. Ethics committees from IMSS and Secretaría de Salud, México, approved the study. Those patients who agreed to participate in this study were required to read and sign an informed consent letter.

This cross-sectional study included all cases of IM (109 patients) and GC (126 patients) available at our sample’s bank that fulfilled inclusion criteria; a similar number of consecutive volunteers (125 healthy controls) attending the blood bank were recruited. Controls were blood donor without any symptom or medication attending the blood bank of the Centro Médico Siglo XXI, IMSS, Mexico City. The exclusion criterion was to have any illness that requires medication. We were unable to strictly match cases and controls by age because the blood donors have to be younger than 45 years of age.

All GC cases were distal cancers, located in the antrum and/or corpus, about 50% type diffuse, 30% type intestinal and 20% mixed; almost 70% of the cases were in stage III or IV.

### Samples

Seven gastric biopsies were taken from each patient, and final diagnosis was defined by the most severe lesion found in any of the biopsies. Blood samples (10 mL) were obtained by venous puncture; peripheral blood mononuclear cells (PBMCs) were purified by centrifugation in a Ficoll-Hypaque density gradient. DNA was isolated from these cells using the QIAamp DNA Blood Mini Kit (QIAgen) and frozen at − 70 °C until tested for SNPs in cytokine promoter regions.

### Determination of *H. pylori* infection

Enzyme-linked immunosorbent assays (ELISA) were performed to detect IgG anti-whole *H. pylori* extract antibodies, and IgG anti-CagA protein antibodies, using ELISA tests validated in our population with a sensitivity of 85% and specificity of 87%, as previously described [9]. *H. pylori* infection was confirmed by histology studies after staining tissues of all biopsies with Giemsa and H&E.

### SNP selection and genotyping

The SNPs to be analyzed were selected according to the following criteria: 1) SNPs were validated by frequency or utilization in the HAPmap Project; 2) SNPs are in the promoter region and have a potential role in transcriptional regulation of the cytokine evaluated (as assessed by the Ensembl browser); 3) SNPs are in IL-4, IL-6, IL-10, TGF-β, TNF-α and IFN-γ promoter regions, in the binding sites of transcription factors that potentially influence transcriptional activity, as reported in Biomart.URL: https://www.ensembl.org/biomart/martview/01e2037218528c76d67d2a5fde286d0c. The polymorphisms *IL-4* –590C/T (rs2243250), *IL-6* –573G/C (rs1800796), *IL-10* –592C/A (rs1800872), *IL-10* –819C/T (rs1800871), *IL-10* –1082A/G (rs1800896), *TGF-β* –509C/T (rs1800469), *TGF-β* –800G/A (rs1800468), *TNF-α* –308G/A (rs1800629), and *IFN-γ* –1615C/T (rs2069705) were selected. A 20-ng sample of genomic DNA was genotyped using predesigned 5′-endonulease assays (Taqman, Applied Biosystems, Waltham, MA) in a 96-well StepOnePlus™ instrument, according to manufacturer’s directions. For quality control purposes, a call rate of 0.99 was used for all samples. Ten percent of the samples studied were randomly selected and reanalyzed to validate the results.

## Statistical analysis

Descriptive variables were analyzed by the Chi-square test; continuous variables were expressed as mean ± standard deviation (SD); and categorical variables were described as percent of the total. Hardy-Weinberg equilibrium models in controls were determined for all SNPs. The risk or protection level for genotypes and alleles was determined as odds ratios (OR) and 95% confidence intervals (95% CI). The association between SNPs and IM or GC was evaluated estimating OR values with multinomial logistic regression models. All statistical analyses were performed using the software Stata/SE v.14 (STATA, Inc., College Station, TX); *P* values < 0.05 were considered as statistically significant.

## Results

Socio-demographic and clinical data of IM and GC patients and control subjects are shown in Table 1. Age, sex, history of alcohol consumption, education level, *H. pylori* infection, and CagA detection were different in IM and GC patient groups with respect to controls. On average, IM and GC patients were older than controls. Also, the proportion of females was higher in IM (65%) and GC (59%) groups than in controls (40%). The proportion of alcohol consumers was higher in controls (70%) than in GC (50%) patients; furthermore, a significant association between alcohol consumption and protection from GC was found (OR = 0.44, 95% CI 0.31–0.64, *P* = 0.001). In IM patients *H. pylori* infection and CagA antibodies were detected in 83.5 and 78%, respectively. In contrast, in the GC group 58.7% were positive for *H. pylori* and 53.2% for CagA.

**Table 1:** General characteristics of the population under study

Variable	Control	Metaplasia	*P*-value	Gastric cancer	*P*-value
Age	*n* = 125	*n* = 109		*n* = 125	
Years (SD)	34 (11.4)	**57.62**	**0.0001**	**59 (12.2)**	**0.0001**
Sex (n, %)	*n* = 125	*n* = 109		*n* = 125	
Female	50 (40)	**71 (65.14)**	**0.0001**	**74 (59.2)**	**0.002**
Male	75 (60)	38 (34.86)		51 (40.8)	
Smoking status (n, %)	*n* = 125	*n* = 109	0.576	*n* = 125	
None	79 (63.2)	65 (59.63)		74 (59.2)	0.51
Smoker	46 (36.8)	44 (40.37)		51 (40.8)	
Alcohol consumption (n, %)	*n* = 125	*n* = 109		*n* = 125	
No	38 (30.52)	66 (60.5)	**0.0001**	62 (49.6)	**0.002**
Regular	**87 (69.6)**	43 (39.45)		63 (50.4)	
Education, years (n, %)	*n* = 125	*n* = 109		*n* = 123	
> 12	55 (44)	1 (0.92)	**0.0001**	10 (8.1)	**0.0001**
10–12	43 (34.4)	7 (6.42)		24 (19.5)	
0–9	25 (20)	**101 (92.66)**		**91 (73.9)**	
*H. pylori* (n, %)	*n* = 123	*n* = 109		*n* = 126	
Negative	69 (56)	18 (16.51)	**0.0001**	52 (41.3)	**0.01**
Positive	54 (44)	**91 (83.49)**		**74 (58.7)**	
CagA (n, %)	*n* = 125	*n* = 109		*n* = 126	
Negative	85 (68)	24 (22.02)	**0.0001**	59 (46.8)	**0.001**
Positive	40 (32)	**85 (77.98)**		**67 (53.2)**	
Family history of gastric cancer (n, %)	*n* = 125			*n* = 125	
No	119 (95.2)	**–**	**–**	**106 (84.8)**	**0.006**
Yes	6 (4.8)	**–**		19 (15.2)	

Values in bold indicate significant differences (*P* < 0.05)

*χ*^2^ test *P*-value

Nine cytokine polymorphisms were genotyped and the association of these SNPs with IM and GC was evaluated by multinomial regression analysis. All SNPs matched with a Hardy-Weinberg equilibrium model in controls. SNPs *TNF*-α -308G/A (rs1800629) and *TGF*-β -800G/A (rs1800468) were not polymorphic in the population under study. No significant association with IM or GC was found with the following SNPs: *IL-4* –590 T/C (rs1800629), *IL-6* –573G/C (rs1800796), *IL-10* –592C/A (rs1800872), *IL-10* –1082A/G (rs1800896), and *IFN-γ* –1615C/T (rs2069705). However, there was a trend toward a significant association of the genotypes CT in the SNP *IL-4* –590 T/C with IM (OR = 0.50, 95% CI 0.24–1.06, *P* = 0.07) and the AA genotype in the IL-10 -592C/A with GC (OR = 0.51, 95% CI 0.24–1.09, *P* = 0.08) (Table 2).

**Table 2:** Association of cytokine gene promoter SNPs with gastroduodenal diseases

IFN-γ -1615C/T (rs2069705)						
	CC^a^	CT		TT		*P*-value^†^
Studied groups (No. of patients)	N (%)	N (%)	OR (95% CI)	N (%)	OR (95% CI)	
Control	54(43.20)	60(48)	1	11(8.80)	1	
Intestinal Metaplasia	8(72.73)	2(18.18)	0.29(0.06-1.50)	1(9.09)	0.64(0.06-6.60)	0.142
Gastric cancer	59(52.21)	49(43.36)	0.81(0.47-1.40)	5(4.42)	0.39(0.12-1.22)	0.225
IL-4-590C/T (rs2243250)						
	CC^a^	CT		TT		*P*-value^†^
Studied groups (No. of patients)	N (%)	N (%)	OR (95% CI)	N (%)	OR (95% CI)	
Control	31 (24.8)	67(53.6)	1	27(21.6)	1	
Intestinal Metaplasia	32(34.4)	29(31.2)	0.50(0.24-1.06)	32(34.4)	1.04(0.47-2.32)	0.004
Gastric cancer	28(22.6)	53(42.74)	0.90(0.47-1.72)	43(34.68)	1.69(0.82-3.48)	0.066
IL-6 -573G/C (rs1800796)						
	GG^a^	GC		CC		*P*-value^†^
Studied groups (No. of patients)	N (%)	N (%)	OR (95% CI)	N (%)	OR (95% CI)	
Control	49(40.16)	58(47.54)	1	15(12.30)	1	
Intestinal Metaplasia	39(41.49)	41(43.62)	0.89(0.46-1.73)	14(14.89)	1.25(0.46-3.37)	0.791
Gastric cancer	49(40.16)	55(45.08)	0.96(0.55-1.68)	18(14.75)	1.49(0.65-3.45)	0.838
IL-10 -592C/A (rs1800872)						
	CC^a^	CA		AA		*P*-value^†^
Studied groups (No. of patients)	N (%)	N (%)	OR (95% CI)	N (%)	OR (95% CI)	
Control	34 (27.20)	61(48.80)	1	30(24.00)	1	
Intestinal Metaplasia	30(28.04)	47(43.93)	0.70(0.34-1.44)	30(28.04)	0.96 (0.42-2.16)	0.715
Gastric cancer	39(31.71)	64(52.03)	0.85(0.47-1.56)	20(16.26)	0.51(0.24-1.09)	0.301
IL-10-819C/T (rs1800871)						
	CC^a^	CT		TT		*P*-value^†^
Studied groups (No. of patients)	N (%)	N (%)	OR (95% CI)	N (%)	OR (95% CI)	
Control	33 (27.05)	60(49.18)	1	29(23.77)	1	
Intestinal Metaplasia	32(32.32)	45(45.45)	0.70(0.34-1.42)	22(22.22)	0.70(0.30-1.63)	0.693
Gastric cancer	44(35.48)	63(50.81)	0.71(0.39-1.29)	17(13.71)	**0.37(0.17-0.81)**	0.093
IL-10 -1082A/G (rs1800896)						
	AA^a^	AG		GG		*P*-value^†^
Studied groups (No. of patients)	N (%)	N (%)	OR (95% CI)	N (%)	OR (95% CI)	
Control	68(55.28)	44(35.77)	1	11(8.94)	1	
Intestinal Metaplasia	62(59.62)	34(32.69)	0.89(0.47-1.69)	8(7.69)	1.14(0.36-3.57)	0.8
Gastric cancer	62(49.60)	54(43.20)	1.34(0.78-2.32)	9(7.20)	1.19(0.44-3.19)	0.477
TGF-β –509C/T (rs1800469)						
	CC^a^	CT		TT		*P*-value^†^
Studied groups (No. of patients)	N (%)	N (%)	OR (95% CI)	N (%)	OR (95% CI)	
Control	25(21.74)	60(52.17)	1	30(26.09)	1	
Intestinal Metaplasia	17(28.80)	23(39.00)	0.52(0.21-1.31)	19(32.20)	0.66(0.24-1.77)	0.25
Gastric cancer	41(34.45)	54(45.38)	**0.51(0.27-0.98)**	24(20.17)	**0.42(0.20-0.91)**	0.091

Comparisons were made using the asymptomatic group as reference

Adjusted by *H. pylori* infection and sex. Values in bold indicate significant differences (*P* < 0.05)

^†^*χ*^2^ test *P*-value

^a^Ancestral genotype was used as reference category

The genotype TT at the position − 819 of the *IL-10* (rs1800871) gene promoter was significantly associated with a reduction of over 60% in the risk for GC (Table 2). Furthermore, the association analysis showed that allele T of rs1800871 was significantly associated with a protection for GC (OR = 0.63, 95% CI 0.43–0.91, *P* = 0.014; power = 0.64).

On the other hand, the CT genotype in the *TGF-β* –509C/T (rs1800469) polymorphism was significantly associated with a reduction of almost 50% in the risk for GC, whereas the TT genotype was strongly associated with a reduction of about 60% for GC (Table 2). In agreement with this result, the allele T of rs1800469 was associated with a decreased risk for GC (OR = 0.64, 95% CI 0.44–0.94, *P* = 0.023; power = 0.99).

## Discussion

The chronic infection by *H. pylori* promotes an inflammatory environment that may disrupts the homeostasis in the gastric mucosa. This unbalanced inflammatory response may lead to increased cell proliferation and transformation, or to DNA damage. The study of genetic variants in proinflammatory and immunosuppressive cytokine genes therefore might help explain why only a small fraction (less than 2%) of individuals infected with *H. pylori* develop GC. This work aimed to evaluate the association of cytokine gene promoter SNPs with IM and GC in a Mexican population.

Regarding the general characteristics of the population studied, we found that alcohol consumption was associated with protection for GC. However, the association between alcohol consumption and GC is still controversial. For instance, some prospective studies have reported an increased risk for GC in alcohol consumers while other studies have reported a lack of association [10–12]. Similarly, meta-analyses studies have reported either an increased risk for GC associated to alcohol consumption or no association [13–15].

The observed frequency of SNPs TNF*-α* − 308 G/A and TGFβ − 800 G/A showed that these SNPs are not polymorphic in the population under study, in accordance with previous reports which have found that *TNF-α* –308G/A is not polymorphic in Mexican nor in Honduran populations [16–18]. On the other hand, the SNP *TGF-β* − 800 G/A has been less studied and our work is the first report in a Mexican population. Our data showed a borderline association of the genotype CT (SNP *IL-4* –590 T/C) with precancerous IM (OR = 0.50, 95% CI 0.23–1.05, *P* = 0.06; Table 2). Other studies have evaluated the association of SNP *IL-4* –590 T/C with precancerous lesions and GC in a Venezuelan and a Chinese populations, but found no significant association of this SNP with GC [19, 20].

Our results showed that subjects carrying the genotype TT in the locus − 509 of the TGF-β gene had a decreased risk for GC (Table 2). Our result is consistent with a recent meta-analysis, which also found that TT genotype at the locus − 509 of the *TGF-β* gene was protective for GC in an Asian population [21], as well as with other studies [22, 23]. In contrast, this TT genotype has been reported as a risk genotype for GC in an Indian and a Chinese population [24, 25]. The transcription factor AP1 containing JunD regulates the expression of TGF-β by binding to the allele C in the locus − 509 of the promoter and repressing its expression [26]. Thus, the TT genotype would affect this mechanism of repression favoring TGF-β overexpression. Guo et al. (2011) reported that TGF-β serum levels were higher in GC patients carrying the TT − 509 genotype than in patients with the CC genotype [27]. Furthermore, other studies have reported an increased expression of TGF-β in gastric tissue and serum from patients with GC, when compared with subjects with a normal gastric mucosa [28, 29]. TGF-β is an immunosuppressive cytokine that inhibits the cell proliferation and controls inflammation [30, 31], which could explain the role of the genotype TT as a protective factor for GC. However, as the disease progresses and, in combination with other cytokines, TGF-β could exert a negative effect inducing the loss of cell polarity, cell migration, and angiogenesis, favoring metastasis [32–34].

In the current study we found that the genotype TT in the locus − 819 of *IL-10* was significantly associated with a decreased risk for GC. In agreement with our findings, a meta-analysis reported that genotype TT in the locus − 819 of the *IL-10* gene was associated with an overall reduced risk for GC in an Asian population [35]. Another meta-analysis covering 73 studies also in Asian populations, found the same association [36]. Conversely, a study including 234 advanced GC patients and 243 controls in a Chinese population showed no significant association of the SNP *IL-10* –819C/T with GC [37]. The mechanism by which the SNP IL-10 -819 C/T could modulate IL-10 expression has not been elucidated, however, a study found that TT genotype was associated with a lower circulating IL-10 expression in comparison with genotypes CT and CC in *Leishmania Braziliensis*-infected patients; in this context, IL-10 was produced by monocytes and CD4 + CD25+ T lymphocytes [38].

In addition, we found trend for significant association of the genotype AA (SNP IL-10 -592C/A) with GC, which was consistent with the results of Sicinschi et al., who reported that the CC genotype was a risk factor for GC in a Mexican population [39]. The functional analysis of SNP − 592 C/A suggested that IL-10 expression is regulated by the binding of Sp1 and Sp3 transcription factors to the upstream region of this polymorphism. One C to A change decreased the inhibitory effect of Sp1/Sp3 complex, favoring IL-10 expression in monocyte, B and T human cell lines [40]. Importantly, authors denoted that the activation or repression of *IL-10* was dependent on the interaction of Sp1 with other transcription factors and the expression levels of these factors.

Haplotype analyses of the IL-10 SNPs have also shown a role in regulating the IL-10 expression. A number of studies have reported that haplotype ATA *(IL-10 − 592A/− 819 T/−1082A)* is related to lower IL-10 promoter transcriptional activity and lower IL-10 production in comparison to the haplotype GCC [41–43]*. H. pylori*-infected patients with chronic gastritis carrying the GCC haplotype exhibited higher IL-10 mucosal expression levels than ATA carriers and this correlated with a higher prevalence of virulent cagA+/vacAs1+ /babA2+ *H pylori* strains [44]. In line with these results, the GCC haplotype was significantly associated with an increased risk for GC in comparison with ATA haplotype in Japanese, Taiwanese and Chinese populations [37, 45, 46]. In addition, elevated serum IL-10 levels have been detected in GC patients when compared with those in controls and high circulating IL-10 levels have been associated with a worse prognosis [47–49]. Taken together, these results suggest that IL-10 –819TT and -592AA genotypes are associated with a decreased risk for GC by maintaining a low expression of IL-10, which could then favor the APC’s access to the tumor cells and the infiltration of CD8 + T lymphocytes [50].

We are aware of some limitations of our study; first, the sample size was not large enough resulting in a decreased power of the study, which probably masked the association of other SNPs with GC. Even though, it allowed us to identify SNPs significantly associated with GC. Additionally, we tested a reduced number of SNPs in cytokine gene promoter regions, which precludes a comprehensive study of the association of SNPs in regions other than promoters with GC. Another limitation of the study is that we did not match by age GC cases and healthy controls. This is because in our institution blood donors have to be less than 45 years of age, whereas GC usually occurs at later ages. Still, we should notice that in Mexico the incidence rate for GC in the population is less than 10 in 100,000, so that the probability that one of these 125 asymptomatic adults develop GC is extremely low.

In summary, our results showed that the SNPs *TGF-β* –509C/T (rs1800469) and *IL-10* –819C/T (rs1800871) were associated with a reduced risk for GC in a Mexican population. Interestingly, the same SNPs, *IL-10* –809 T/C and TGF-β –509 T/C, were associated with an increased risk for cervical cancer in a Mexican and Asian population [36, 51], which is consistent with the biological differences in the natural history of cervical and gastric cancer, specifically the role of inflammatory mediators in each pathology. Further studies are required to evaluate the role of these SNPs in regulating the expression of TGF-β and IL-10 during GC progression.

## Abbreviations

GC: Gastric cancer
IM: Intestinal metaplasia
SNP: Single Nucleotide Polymorphism
